# Participatory health research with migrants: Opportunities, challenges, and way forwards

**DOI:** 10.1111/hex.13201

**Published:** 2021-02-02

**Authors:** Maria Roura, Sonia Dias, Joseph W. LeMaster, Anne MacFarlane

**Affiliations:** ^1^ School of Public Health University College Cork Cork Ireland; ^2^ NOVA National School of Public Health, Public Health Research Center Universidade NOVA de Lisboa & Comprehensive Health Research Center (CHRC) Lisboa Portugal; ^3^ Kansas University Medical School Kansas City KS; ^4^ School of Medicine Limerick Limerick Ireland; ^5^ Health Research Institute University of Limerick Limerick Ireland

**Keywords:** community involvement, community‐based participatory research, Transients and migrants

## Abstract

**Context:**

Migration is one of the most politically pressing issues of the 21st century but migrant health remains an under‐researched area. The International Collaboration for Participatory Health Research (ICPHR) working group on migration developed this position statement to address opportunities and challenges in relation to migrant health. It aims to contribute to a shift from a deficit model that sees migrants as passively affected by policies to their reconceptualization as citizens who are engaged in the co‐creation of solutions.

**Methods:**

This paper examines the opportunities and challenges posed by the use of PHR with migrants. It draws on a broad literature to provide examples of successful PHR with migrants and highlights critical issues for consideration.

**Findings:**

Successful initiatives illustrate the value of engaging migrants in the definition of the research agenda, the design and implementation of health interventions, the identification of health‐protective factors and the operationalization and validation of indicators to monitor progress. Within increasingly *super diverse* contexts, fragmented community landscapes that are not necessarily constructed along ethnicity traits, inadequate structures of representation, local tensions and operational barriers can hamper meaningful PHR with migrants.

**Conclusion:**

For each research context, it is essential to gauge the ‘optimal’ level and type of participation that is more likely to leverage migrants’ empowerment. The development of Monitoring and Evaluation tools and methodological strategies to manage inter‐stakeholder discrepancies and knowledge translation gaps are steps in this direction.

**Patient or public contribution:**

This paper draws from contributions of migrant populations and other stakeholders to policymaking.

## 1 INTRODUCTION

Migration has become one of the most politically pressing issues of the 21st century. It is a diverse experience, with potential for both positive and negative impacts for individuals and societies as a whole.^1^

There is no standardized way to define ‘who is a migrant’.^2^ For the purpose of this paper, we consider as migrant ‘*any person who is moving or has moved across an international border or within a State away from his/her habitual place of residence, regardless of the person's legal status, whether the movement is voluntary or involuntary, what the causes for the movement are and what the length of the stay is’*.^3^ The vast majority of migrants in the world are migrant workers but the numbers of refugees and people displaced by conflict, natural disasters and climate change are at their highest levels, representing 10% of all migrants who move between countries.^4^, ^5^ This underscores the importance of addressing the health of migrants as a part of the global health‐for‐all agenda.

Acknowledging the essential relationship between good health and successful migration, the World Health Organization (WHO) Member States adopted the 2008 World Health Assembly Resolution on the health of migrants (WHA.61.17) and launched an Strategy and Action Plan for Refugee and Migrant Health in 2016.^6^ In addition, the Colombo Statement, which was endorsed by 19 Ministers and government representatives in 2017, affirmed that migrants should be active stakeholders in programme planning and decision making.^7^ Still, migrant health remains an under‐researched area in global health and has received insufficient attention by health system planners.

Although migrants are sometimes healthier than the host population on arrival,^8^, ^9^ there is evidence of health disparities between some migrants and their host populations and a growing awareness that this is linked to the negative impacts of the broader social determinants of health (SDH).^8^, ^12^, ^13^ This includes a pattern of exclusion whereby migrants are under‐represented in health‐care decision‐making fora for citizens.^14^, ^15^

Appropriate methodological approaches are needed to respond to the challenges associated with contemporary migration, mobility and health.^16^ Participatory Health Research (PHR) is a research paradigm that has potential to address opportunities and challenges in relation to migrant health. The goal of PHR is ‘to maximize the participation of those whose life or work is the subject of the research in all stages of the research process, including the formulation of the research question and aim, the development of a research design, the selection of appropriate methods for data collection and analysis, the implementation of the research, the interpretation of the results, and the dissemination of the findings’.^17^ It is guided by ethical principles to reflect its underpinning values including mutual respect, equality and inclusion.^18^ In PHR, relationship building and the value of sustained partnerships throughout a project from question identification to result dissemination is of paramount importance.^19^Grounded on the work of Paulo Freire, the ultimate aim of PHR is to catalyse broad societal transformations for a more fair allocation of resources.^20^, ^21^ To this end, the entire process of PHR is conceived to leverage joint societal transformation and transcend the scope of the specific objectives of a particular project. The underlying assumption is that engaging research participants as co‐producers of new knowledge fosters their ownership over the research outcomes, which can then serve to articulate and legitimate political claims to address the social determinants of health.

## 2 METHODS

The International Collaboration for Participatory Health Research (ICPHR) working group on migration developed this position statement to address the opportunities and the challenges posed by the use of this research paradigm in migrant health research. It draws on a broad literature to provide examples of successful PHR with migrants and highlight critical issues for consideration. Members of the ICPHR provided comments on a draft version of this paper which was distributed at the ICPHR 10th Annual Working Meeting at Morgan State University in Baltimore, Maryland, USA (21‐22 June 2019).

## 3 FINDINGS

### 3.1 Opportunities

#### 3.1.1 Define the research agenda

Most of the published academic research that has so far been conducted in the field of migrant health represents the perspectives of high‐income destination countries and focuses on migrant‐specific diseases with a particular emphasis on communicable diseases and the mental health of refugees.^4^ This focus on differences between migrants versus the local populations has led researchers to overlook some of the most common health problems that affect migrants, which are often similar to those affecting the host population.^11^, ^12^, ^22^

Concepts of civic responsibility and participation^21^, ^23^, ^24^, ^25^ emphasize migrants’ *right* to shape the research agenda so research efforts address what migrants perceive as priority needs.^26^ Decisive endorsement of the principle of participation is reflected in the increasing requirements by research funders and renewed international commitments to meaningfully involve the public and patients in health research,^27^, ^28^, ^29^ including migrants.^7^ Still, to date, the research priorities in migrant health have been primarily driven by the interests of academics, policymakers and clinicians^10^ with infrequent inclusion of migrants in research prioritization processes.^12^, ^15^

Setting priorities for research is a complex process, and there is general consensus that there can be no *best practice*, because of the contextual differences between individual priority setting exercises.^30^ PHR, with its focus on incorporating different perspectives to foster mutual learning and deliberation, can be helpful to structure democratic dialogues amongst migrant and other stakeholders and develop a shared vision for research priorities. Previous participatory research initiatives with migrants,^31^ including use of the *World Café method,* have proved to be effective for research prioritization with migrants in Ireland and the USA.^26^

#### 3.1.2 Inform the design and implementation of adequate health interventions

Epidemiological research has shown that some migrants are more affected by communicable diseases, occupational health hazards, injuries and maternal and child health problems than the local population.^11^, ^32^ Some groups are particularly vulnerable including unaccompanied children, victims of trafficking or torture, asylum seekers and migrants in irregular status or in detention centres.^10^, ^33^ However, migrants are often not granted equitable access to health services, and these may not have sufficient capacity to manage their needs in a culturally, linguistically or clinically adequate manner.^2^, ^34^ Widely documented barriers to accessing quality health‐care services include: lack of entitlements, fear of losing employment or residency if affected by certain medical conditions,^7^, ^35^ administrative hurdles and communication barriers.^36^ In addition, certain health interventions^37^ may violate individual rights or exacerbate discrimination, for example, when migrants are screened for infectious diseases without adequate referral to treatment when needed.^7^, ^38^, ^39^ The provision of sensitive services is thus essential to respond adequately to the diverse needs of increasingly heterogeneous populations.^10^, ^37^, ^40^ However, most interventions and policies are based on data derived from the general population and do not respond to the needs of migrants.^41^ Where evidence is lacking, PHR can be a good strategy to fill that gap and pave the way to develop more effective interventions and policies.

PHR acknowledges the importance of experiential, practical, emotional and intuitive sources of knowledge. It builds on the *insider* perspectives and direct knowledge acquired by the people living with the health problem under study,^42^ who are considered *experts by experience*.^17^ The multiple ways of knowing that are inherent to PHR can yield the holistic and nuanced understanding that is required to bridge different explanatory models of disease. This can prevent ethnocentric biases in the development of health interventions.

Previous successful PHR initiatives to involve migrants in the adaptation of health services include: the development and implementation of guidelines to improve communication in cross‐cultural consultations in 4 European countries,^43^ the co‐production of a breast screening video by Asian migrant women in the UK,^44^ the co‐design of a child obesity intervention,^45^ a diabetes prevention programme with Sikh Asian Indians in New York,^46^ the development of a mental health intervention with Bangladeshi women in the Bronx,^47^ an HIV prevention programme with Latinos in the USA^48^ and the development of a computer‐assisted safer sex intervention.^49^ These initiatives involved migrants in the design and implementation of the initiative from start to finish and at multiple levels within health‐care organizations and processes. They suggest that migrants *can* be involved effectively in participatory research and decision making to adapt health‐care services and interventions, so they are relevant, respectful, responsive to their lived experiences and aligned with their needs.^50^, ^51^, ^52^, ^53^

#### 3.1.3 Tackle the Social Determinants of Health (SDH)

High‐quality care for migrants cannot be addressed by health systems alone. Migrants from low to high‐income countries are often marginalized^22^ and exposed to social, occupational and economic conditions that have detrimental effects on their health.^7^, ^54^, ^55^ The death of migrants during their migration journey is a tragic illustration of the vulnerabilities that affect migrants at different stages of a migration process that often entails unsafe travel, poor nutrition, psychosocial stressors and harsh living and working conditions.^7^

A comprehensive response to the needs of migrants requires health systems to engage with other key sectors such as welfare, housing, education and legal protection.^56^, ^57^ While the importance of the SDH is widely recognized,^7^, ^11^ the role of public policies beyond the health sector continues to be overlooked in migrant health policies.^58^ In turn, the SDH agenda has been criticized for adopting a ‘colour‐blind’ approach that presumes that an improvement of socio‐economic conditions will have a homogeneous impact on the health of different ethnic groups.^59^ Indications that migrants do not fully reap the expected health benefits associated with improved material conditions point to gaps in our understanding of how ethnicity and socio‐economic status intersect with other SDH (eg racism) to influence migrants’ health^8^ and call for a more explicit acknowledgement of structural and historical factors as has been highlighted by *critical race* ^60^ and *intersectionality* scholars.^61^

Because it is locally situated in the everyday life of research participants, PHR enables the contextualization of individuals’ local knowledge and lived experiences across the different layers of the *social ecology*. This means that besides accounting for the individual and family factors that influence health, a broad range of community level and broader structural/historical factors must also be considered, including neighbourhood characteristics and the ethnically patterned unequal distribution of resources and power. McElfish et al^62^, for example, report the use of PHR to engage a displaced Marshallese community in Arkansas USA using a sociological lens to identify organizational, community and policy barriers that constrained self‐management efforts by community members affected by Type 2 Diabetes.

Participatory research should lead to action and ensure that the benefits of the research are shared with relevant local actors. Enabling diverse stakeholders to learn from each other and plan together can yield fresh ideas about the conditions that are necessary to sustain optimum health at each level of the social ecology and the policy initiatives that can produce these conditions. Previous work with ethnic minorities suggests that PHR can effectively promote broader level societal change. In Kansas City, Missouri, for example, a participatory initiative with Black Americans leveraged positive change in schools, churches, the media and the private sector.^63^ In London, the participation of migrant women in a breast screening promotion project was reported to be an empowering experience that challenged the view of migrant women as homogeneous and powerless victims.^44^

#### 3.1.4 Identify health‐protective factors

Despite the importance of addressing migrants’ vulnerabilities using a SDH approach, it can be harmful to assume that the health of migrants is always poor when compared to the host population.^9^ The focus on vulnerability can obscure evidence showing migration as a positive experience for many and the fact that many migrants are young, fit and healthy.^7^ Still, migrants are often framed as carriers of disease, difficult health‐care users, poorly compliant^64^ and, ultimately, a burden to health systems and societies at large.^65^ Worryingly, the argument that diseases travel in migrant's blood is recurrently used by anti‐migrant political leaders to advance their political agenda.^8^, ^66^

A better understanding of what make some migrant populations healthier could contribute to breaking down harmful stereotypes about migrants^8^ and provide valuable clues about how to preserve the health of migrants and populations as a whole. Whereas conventional research tends to focus on the deficits of vulnerable people, PHR builds on their *strengths* ^17^ and their own accounts of *what goes on* in their everyday life. This familiarity with the environment can potentially unveil how health‐protective assets are acquired and maintained over time, and what are the contextual conditions that enable or constrain this. PHR could help to unravel how psychological resources (eg positive identity, confidence, optimism, connectedness) are embedded within social structures (eg social hierarchies at work, at home, in public spaces). This would further our understanding of the contingent conditions that foster/hinder health for different people in different contexts, broadening the current focus on individuals’ behaviour and psychological skills by placing individuals’ choices in context.

#### 3.1.5 Operationalize and validate indicators to monitor progress

The evidence base and action to address health inequities affecting migrants cannot be furthered without robust monitoring frameworks grounded on reliable measures and the definition of concrete indicators against which actionable goals and targets can be set. These indicators should transcend disease‐based surveillance approaches to also include the broader social determinants of health^67^ and adequately capture the constructs that they are intended to measure (ie be valid and reliable). International recommendations advise national governments to review existing monitoring mechanisms across the health and development sectors to incorporate migrant health‐related variables and engage in target‐setting processes.^7^ Ideally, a common set of internationally comparable indicators should be employed. However, there is no standardization/harmonization and the validity of some of the most commonly employed indicators in migrant health research has been questioned. A long tradition of *acculturation r*esearch, for example, has extensively employed one‐dimensional definitions of *ethnicity* that fail to recognize that identities are diverse and neither stable nor unconfounded.^68^ Similarly, subjective measures of self‐rated health are often used as an indicator of health status^69^ although the meaning of *excellent*, *good, fair* or *poor* health differs across populations.^70^, ^71^ Finally, the indicators of socio‐economic status that are employed in most SDH studies (eg occupational class, income, education) do not measure accurately the economic status of migrants, whose personal income often fluctuates and is not always associated with educational levels.^68^ These indicators also miss less tangible dimensions of subjective social status^72^ (eg participation, prestige, integration in social networks) that predict health outcomes independently of traditional indicators of socio‐economic status.^73^

PHR can account for the subjective, dynamic, multifaceted and contextual nature of the indicators that are commonly used in migrant health research, helping to unpack their social significance. Previous research on ethnic and migrant health in Ireland illustrates the potential of PHR to operationalize key constructs in relation to health information systems.^74^ The co‐creation of a new instrument to measure mental health with Bangladeshi women in the Bronx, New York^75^ and the operationalization of the concept of *well‐being* by Moroccan migrants in Spain^76^ show that PHR can broaden and deepen our understanding of how to measure multifaceted concepts in epidemiological research, while building collaborative capacity to ensure adequate design and usage of monitoring instruments. This is essential to track health systems performance and the impact of diverse policies on migrant health.

Having presented successful examples of PHR in which the involvement of migrants was feasible and impactful, we next consider key challenges and potential strategies to overcome them.

### 3.2 CHALLENGES

#### 3.2.1 Power dynamics

Conducting PHR with migrants is not exempt of challenges some of which are common to all PHR in general. Frequently reported barriers in PHR that can impact on PHR with migrants include conflicts amongst participants, often because of issues related to sharing power and the distribution of resources amongst stakeholders.^17^ The ‘fall back into dichotomies of power’ or ‘tyranny of participation’ whereby the nature of power dynamics within and amongst stakeholder groups is overlooked, and only the narrow spectrum of interests of the most powerful/vocal is considered, is another frequently highlighted challenge of participatory research.^17^, ^77^

Other concerns are the modest impact of participatory research in terms of specific actions bringing about societal change,^78^ mostly because of the limited control that PHR participants often have over key political decisions.^79^ The assumption that participants will have the necessary time available for contributions, the criteria used to economically compensate some contributors but not others, the amount of the economic rewards provided, the mismatch of expectations, accountability issues, different communication styles/ perceptions of time and the limitations posed on researchers’ autonomy are other of the challenges highlighted.^78^, ^80^, ^81^ Ethical concerns may arise in relation to multiple (and at times conflicting) roles assumed by researchers and organizational stakeholders (eg as fundraisers and resource allocators).^82^, ^83^ Finally, the uneasy confrontation of lay researchers with managerial procedures and fixed time lines may place additional strain over the smooth implementation of PHR.^84^

#### 3.2.2 Definition of ‘migrant communities’

Amidst the conceptual and practical difficulty of defining who is a ‘migrant’, it is also difficult to define ‘migrant communities’ and their ‘representatives’. Social scientists have long contested idealized notions of ‘communities’.^85^ The assumption that these are constructed primarily around ethnicity is hotly critiqued by ethnicity scholars as inadequately linked to pre‐conceived ideas of homogeneity and identity.^86^ The over‐culturalization of the concept – it is argued – leads to a ‘collective image of communion premised on a shared culture’ that fails to capture the actual context of real‐world settings. The loose use of the concept as ‘black box’ ^87^ is problematic because ‘the *community* becomes too easily an explanation, as opposed to something to be explained’.^87^, ^88^

On a more practical side, migrant populations are often very mobile, with frequent remigration to other countries, regions or neighbourhoods. Migrant communities are often dispersed in transnational networks and materialized ‘online’, as opposed to being tied to a physical location. This transient situation hampers the establishment of settled ‘communities’ with relative durable boundaries with which to conduct research. This is especially the case in countries that are unwilling to support the formation and maintenance of civil society structures or where governments and philanthropists favour to financially support local charities focused on providing basic services to vulnerable migrants, as opposed to strengthening the rights of migrants within the core infrastructure of civil society. As a result of these factors, the associational landscape of migrant organizations can be thin and fragmented.

#### 3.2.3 Representativity

The absence of formal, physically bounded migrant communities often leads to research partnerships being established with organizations that provide services to migrants, as a proxy for migrants themselves.^89^ While there are positive examples, it is prudent to be aware of potential limitations in terms of truly representing migrants’ views. This is particularly worrying in contexts where assimilationists policies or cultures prevail and where the fundamental principles underlying PHR are not necessarily endorsed by migrant ‘representatives’. A charity worker performing as ‘community representative’, for example, may not endorse ideas around migrant empowerment and see migrants as passive recipients of charity that should ‘adapt’ to the host society, as opposed to active contributors to enrich a multi‐cultural society.

Even where ‘migrant communities’ exist in the form of established migrant organizations, we cannot assume that these will always represent the interests of ‘migrants’ as a whole. Early calls from development scholars warned that non‐participatory, ‘top‐down’ assumptions made by international development programmes during the 20th century could be repeated in the health field.^23^ As a matter of fact, an individuals’ role as ‘community representative’ may confer him (or her) an increased control over how resources are used/distributed and serve to reinforce the power of community‐based elites.^84^, ^90^

As noted by Wright, PHR is not universally nor categorically ‘better’ than other forms of research.^17^ Understanding migrant associations’ landscape, their role and functions, and – importantly – their linkages with the broader communities and the State, is crucial to decide the type and level of participation that suits each specific research setting. Key questions to ask since the outset include: what type of community organizations exist?, What type of activities they conduct? and Who participates in them and why?.^23^ This should be useful to assess the extent to which particular groups of migrants (eg newcomers, irregulars, asylum seekers, trafficked persons) are represented, and what should be done to ensure their views are also taken into account.

#### 3.2.4 Local tensions

The assumed existence of ‘migrant communities’ willing to work together for a common goal is further challenged in increasingly *super diverse* contexts^91^, ^92^ in which different migrant groups may not necessarily share the same interests or maybe share some, but compete for others. The high rate of *Brexit* voters amongst long established migrant communities in the UK is an illustrative example that challenges the assumption that all migrants share a common goal.^93^ Because the more recently arrived migrants often lack structures for effective representation, their views are less likely to be accounted for, and not necessarily fall under the umbrella of ‘migrant’ interests, as voiced by the most organized groups.^85^ The coexistence of shared and competing interests is also prevalent amongst migrants ‘belonging’ to the same ethnic group, because ‘identity and interest are not insoluble’,^94^ and different sub‐groups are likely to hold – at least some – diverging interest and views (eg youth, women). In contrast with the ideal of cohesive communities, the everyday spaces of neighbourhoods are in fact often characterized by tensions, fragmentation, competition and conflict. Idealized notions of ‘community’ can thus serve to actually mask and even reinforce wider structural inequities, which is clearly at odds with the principles underlying PHR. It is thus essential to reconceptualize the concept of migrant communities in more fluid terms (eg not necessarily constructed along ethnicity traits), acknowledge the existence of conflict, as well as the potential inadequacy of organized structures of representation that may exist. The likely rise of conflict of interests needs to be expected, assessed, monitored and disclosed.

In this context, it becomes crucial to adopt a balanced approach that eschews the ‘idyll of community’ critiqued by ethnicity scholars,^95^ but also an exclusive focus on conflict and local tensions. This will help to demystify the role played by communities and its representatives while at the same time help PHR investigators to focus on identifying potential niches of shared interests and aspirations around which common efforts can be articulated.^96^

Formative research following the principles of PHR can be useful to assess whether and how heterogeneous populations and stakeholders may cooperate successfully, by putting aside differences and work towards a common goal that may actually produce a shared ‘sense of community’. Where this is unlikely to be the case, it will be crucial to acknowledge that less or a different kind of participation may – in fact – be the ‘optimal’ level or type of participation for a particular research context.

#### 3.2.5 Operational barriers

At programmatic and implementation level, there are commonly reported challenges that need to be addressed. Language barriers frequently lead to the exclusion of migrants who do not speak the host society language(s), who are already amongst the most socially excluded. This has major implications in terms of equity. The use of visual and culturally adaptable Participatory Learning and Action research techniques^12^, ^52^ with the collaboration of trained interpreters and peer researchers can be an effective way to overcome these.^50^, ^97^ The involvement of peer researchers, however, can lead to blurred personal and project boundaries and requires an ethical and reflective approach.^98^

Other ethical issues related to PHR with migrants include negative consequences from taking part in research, as this could put migrant populations at risk of greater marginalization into even greater peril. Ensuring that informed consent procedures truly inform migrants of both the benefits and potential risks of participation becomes essential here. This may be hard to achieve when the invitation to participate comes from organizations that provide social services to prospective research participants. Careful decisions need to be taken over the most adequate compensation and other types of support to be provided to participants, taking into consideration the characteristics and risks of each particular context. A number of resources are available to guide such decisions in accordance with the ethical principles of PHR.^18^

Another common challenge is related to other PHR stakeholders’ priorities. For example, academics are often committed to traditional (non‐PHR) methods and may feel pressured to quickly publish the evidence in high‐impact scientific journals. Policymakers or industry stakeholders may be resistant to research findings that challenge their assumptions, values, attitudes, or practices or lack the commitment (or power) to respond to the specific concerns expressed by migrants.^81^ Divergence and controversy arise while achieving a compromise to meaningful consensus, which implies negotiation between conflicting interests. Ideally, such a process should help actors to reorient and expand how they define the ‘problem’ under discussion, considering their multiple perspectives of analysis of the project and different interpretations of its successes/failures. However, in practice, this is not always the case, and inequalities between negotiating actors may end up favouring those who are most powerful.^99^, ^100^

The above challenges illustrate the importance of maintaining a high standard of quality and building the empirical evidence about the value of PHR. In this process, it is important to avoid tokenistic approaches where participatory claims are used as a strategy to implement already designed policies rather than to provide spaces for populations to advocate for transformative initiatives. Participatory processes should be described in a transparent and self‐critical manner with a comprehensive account of the achievements but also the challenges and limitations faced.^101^ Several points should be considered to advance in this direction. First, regular monitoring and evaluation (M&E) exercises within PHR partnerships should gather stakeholders’ perspectives of how things are progressing, and when and how adjustments shall be needed. Robust M&E frameworks are urgently needed to guide these processes, with particular attention to power dynamics that may hinder transformative participation dynamics.^100^ In a decisive step in this direction, a M&E working group established within the ICPHR is already drawing from various conceptual frameworks and the views of global PHR practitioners to identify relevant domains, indicators and questions to be asked.^102^ Second, guidance is needed on how to recruit, engage and create fruitful inter‐stakeholder alliances in this particular field of research. A prerequisite to shared decision making is that partnerships and coalitions are established with inter‐sectoral stakeholders.^17^, ^103^ The many different kinds of potential interactive spaces for participation should be considered,^104^ including those established by the State, academics or by migrant populations themselves. In addition, innovative methodological strategies are needed to identify and address conflicting priorities amongst different actors within the broader contexts in which research takes place.^101^ The use of arts is an interesting avenue to explore in this direction.^105^ Finally, it is important to manage expectations and make it clear at the outset of projects that societal change may not be achieved because of external constraints. While the commitment is *towards* action rather than *guaranteeing* action, explicit and proactive steps should be taken to foster the involvement of migrant partners in collaborative knowledge translation activities to reduce the knowledge‐to‐practice gap. Bidirectional mentoring between academic and under‐represented group**s**, for example, is a promising approach that has already been successfully applied with ethnic minorities.^106^ All these actions shall be helpful to prevent tokenism and co‐optation in this field of research.

## 4 CONCLUSION

PHR presents an opportunity to contribute to generating new knowledge about migrants and their health, by bringing together stakeholders who do not usually meet each other in partnerships for research and policymaking. It can potentially contribute to a paradigm shift, from a pathogenic *deficit model* that sees migrants as passively affected by policies to their reconceptualization as creative, inspiring and actively engaged citizens in search of solutions.^8^ This is important to counter the toxic discourse that migrants are a burden to local societies and can help to break down stereotypes by highlighting their positive contribution to social and economic prosperity.^1^, ^5^, ^11^

This paper has emphasized the relevance of PHR in the field of migrant health research, providing an alternative approach to address the current challenges in health research and tackle health inequities. PHR is not, however, a panacea, and there are specific challenges in enacting meaningful and impactful projects in this field. The ultimate distinctiveness and added value of PHR rests in its potential to catalyse real‐world action for greater social justice. Supportive policy environments are essential for this potential to be realized. A genuine progress of PHR with migrants calls for meaningful engagement of inter‐sectoral and ‘whole’ governmental policymakers. In this process, it becomes particularly crucial to grasp – for each particular research context – what is the ‘optimal’ level and type of participation that is more likely to leverage migrants’ empowerment so they can better advocate for their voices to be heard, and their rights to be addressed.

At a time where the case for participatory research is gaining momentum, it becomes crucial to encourage and support critical scholarship and reflective, ethical practice,^18^ not only in the application of PHR with migrants, but also in better understanding the nuances of the approach, so that it can truly live up to its potential. The development of M&E frameworks and methodological strategies to manage inter‐stakeholder discrepancies and knowledge translation gaps are important steps in this direction.

## CONFLICT OF INTEREST

The authors declare no conflict of interest.

## Data Availability

Data sharing is not applicable to this article as no new data were created nor analysed in this study.
